# Anti-IFN-γ Autoantibody Syndrome Presenting with Disseminated Nontuberculous Mycobacteria Infections: A Case Series of Therapeutic Implications and Review of Literature

**DOI:** 10.3390/tropicalmed10070202

**Published:** 2025-07-21

**Authors:** Brooke Cheng, Barinder Bajwa, Seungwon Choi, Hannah Martin, Tyson Miao, Denise Werry, Michael Perlman, Yazdan Mirzanejad

**Affiliations:** 1 Department of Medicine, University of British Columbia, 2775 Laurel Street, Vancouver, BC V5Z 1M9, Canada; 2 Faculty of Medicine, University of British Columbia, 317-2194 Health Sciences Mall, Vancouver, BC V6T 1Z3, Canada; bbajwa@student.ubc.ca (B.B.); martin46@student.ubc.ca (H.M.); 3 Department of Dermatology and Skin Science, University of British Columbia, 835 West 10th Ave., Vancouver, BC V5Z 4E8, Canada; 4 Department of Medicine, Queen’s University, 94 Stuart Street, Kingston, ON K7L 3N6, Canada; choi.s@queensu.ca; 5 Division of Infectious Diseases, Surrey Memorial Hospital, 13750 96 Ave., Surrey, BC V3V 1Z2, Canada; tyson.miao@fraserhealth.ca (T.M.); denise.werry@fraserhealth.ca (D.W.); yazdan.mirzanejad@ubc.ca (Y.M.); 6 Pharmacy, Surrey Memorial Hospital, 13750 96 Ave., Surrey, BC V3V 1Z2, Canada; 7 Division of General Internal Medicine, University of British Columbia, 5913-1081 Burrard Street, Vancouver, BC V6Z 1Y6, Canada; permi@telus.net; 8 Division of Infectious Diseases, University of British Columbia, 2733 Heather Street, Vancouver, BC V5Z 3J5, Canada

**Keywords:** mycobacterium avium complex, nontuberculous mycobacteria, rituximab, interferon gamma, immune deficiency, case series

## Abstract

Anticytokine autoantibodies (AAbs), particularly anti-interferon-gamma (anti-IFN-γ) AAbs, disrupt cytokine functions, leading to infections, autoimmune-like diseases, and conditions resembling interleukin-12 (IL-12)/IFN-γ pathway defects. Advances in genetic testing have clarified overlaps between autoinflammatory, autoimmune disorders, and primary immunodeficiencies but reveal complex phenotypes and pathways. While these insights deepen our understanding of immune mechanisms, they also complicate diagnosis and treatment, with limited options for IFN-γ deficiencies caused by genetic mutations. The adult-onset immunodeficiency with disseminated lymphadenitis due to nontuberculous mycobacteria (NTM) and other opportunistic infections has been linked to high levels of anti-IFN-γ AAbs. This syndrome, initially identified in HIV-negative Asian patients, frequently affects individuals of Asian descent and may be associated with specific human leukocyte antigen (HLA) alleles. The presence of neutralizing anti-IFN-γ AAbs impairs the IFN-γ-dependent immune response, likely contributing to the persistent NTM infection. This study underscores the potential for late-onset anti-IFN-γ AAb syndrome to manifest with disseminated NTM (dNTM) infections, highlights the importance of timely diagnosis and considers rituximab as a potential therapeutic option.

## 1. Introduction

**Figure 1:**
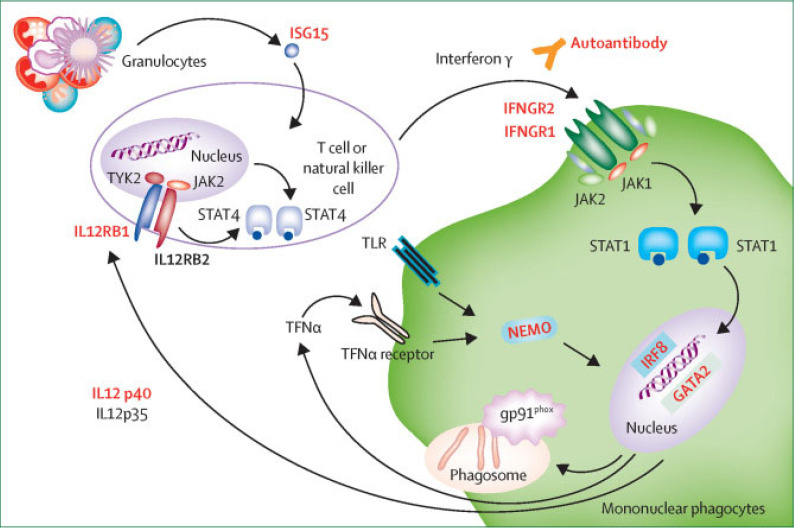
Host defense mechanisms against non-tuberculous mycobacteria (NTM). Defects leading to disseminated NTM infection are shown in red. ISG15, interferon-stimulated gene 15; IFNGR, interferon-gamma receptor; TYK, tyrosine kinase; JAK, Janus kinase; STAT, signal transducer and activator of transcription; GATA, transcription factor implicated in early haemopoietic, lymphatic, and vascular development; NEMO, nuclear factor kappa-light-chain-enhancer of activated B cells essential modulator; IL, interleukin; TFN, tumor necrosis factor; TLR, toll-like receptors. Reprinted with permission from Wu et al. [15]. Copyright 2015, Elsevier Ltd.

Infectious complications due to anti-interferon-gamma autoantibody (anti-IFN-γ AAb) positivity have become increasingly recognized over the last 20 years [1,2]. Since their identification in 2004, anti-IFN-γ AAbs have emerged as a significant global health issue, especially due to susceptibility to disseminated nontuberculous mycobacteria (dNTM) infections. These cases, most prevalent in Southeast Asia but seen globally, are often misdiagnosed due to symptom overlap with *Mycobacterium tuberculosis*. QuantiFERON-TB indeterminate results may occur in the presence of anti-IFN-γ AAbs, further complicating the diagnostic process. Treatment typically involves prolonged antimicrobial therapy, but new immunotherapies including rituximab, cyclophosphamide, and combination treatments like R-CHOP show promise, particularly in refractory cases [3]. IFN-γ is essential for connecting myeloid and lymphoid immune pathways, particularly for macrophage activation. Disruption in these pathways predisposes individuals to various infections, with nontuberculous mycobacteria (NTM) infections being most common. Two studies reported that approximately 80% of patients with dNTM infections and normal CD4 lymphocyte counts tested positive for anti-IFN-γ AAbs [3,4]. It has been suggested that anti-IFN-γ AAbs often appear before infection, though their triggers are uncertain and may involve molecular mimicry and prior immune responses [5]. Elevated levels of these AAbs disrupt IFN-γ signaling, impeding macrophage activation, cytokine production, and JAK-STAT pathway function (Figure 1), all of which are crucial for combating intracellular infections. Experimental models showed that the absence of IFN-γR in mice provides resistance to lipopolysaccharide toxicity by reducing tumor necrosis factor (TNF) synthesis, lowering CD14 expression on macrophages, and diminishing inflammatory responses [2]. However, in chronic infections like NTM and fungi (e.g., *Cryptococcus* and *H. capsulatum*), IFN-γ pathway defects prevent inflammatory signaling and pathogen clearance. IFN-γ has an essential role in mycobacterial defense and potential immune-based therapies for these infections [6]. Diagnosis is often challenging and delayed, with median times of one to 1.6 years in reported cases [7,8,9]. Lymph nodes, bone, and lungs are common sites of infection [10]. In one study, 80% of anti-IFN-γ AAb-positive patients with opportunistic infections presented with peripheral lymphadenopathy, leukocytosis, lymphopenia, and elevated C-reactive protein (CRP) [9]. Nonspecific symptoms can lead to misdiagnoses, such as malignancy or *Mycobacterium tuberculosis*, especially when acid-fast bacilli are identified in samples [7,9,11]. Lymph node histopathology may also mimic lymphoma, IgG4-related disease, or Castleman disease [11].

Under normal circumstances, IL-12 activates CD4+ T cells and stimulates IFN-γ production, leading to further activation of phagocytes through a positive feedback loop. However, anti-IFN-γ AAbs impedes IFN-γ binding, halting this loop and increasing infection endurance and resistance to intra-cellular demise [5,12,13,14]. The diagnosis of anti-IFN-γ AAb syndrome involves assays such as ELISA and flow cytometry to assess the binding and neutralizing effects of these AAbs. Treatment remains complex due to the absence of standardized protocols, although antimicrobial therapies and immunomodulatory agents, particularly rituximab, have demonstrated potential efficacy. Alternatives like cyclophosphamide and daratumumab are also considered for refractory cases. The need for further research is emphasized to develop effective treatments and enhance the understanding of this immunodeficiency, especially in Asian populations where it is more prevalent [5]. Here, we present two cases who were diagnosed and treated with rituximab following poor clinical response to an extended duration of antimicrobial therapy.

## 2. Clinical Case Summaries

**Figure 2:**
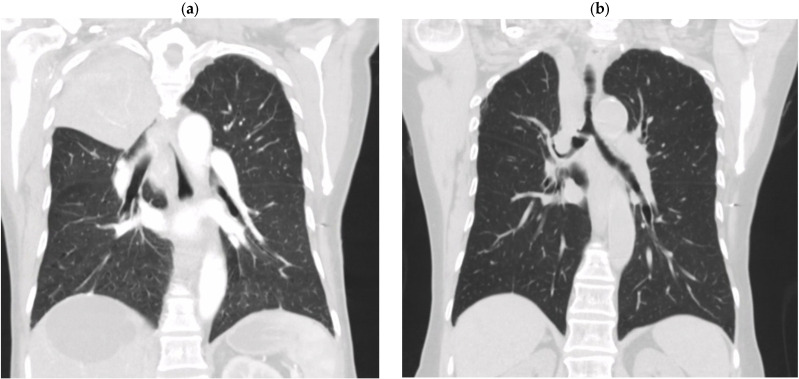
Evolution of chest CT imaging in Case 1: (**a**) before treatment—December 2021, (**b**) after treatment—December 2023. Interval improvement of dense consolidation in the right upper lobe.

Case 1: A 74-year-old Southeast Asian female, originally from Hong Kong, presented with a 7-month history of a productive cough, occasional hemoptysis, constitutional symptoms, and weakness. Past medical history was notable for remote bilateral mastectomies for breast cancer, paroxysmal atrial fibrillation with dual lead permanent pacemaker for sick sinus syndrome and gastroesophageal reflux disease (GERD). Laboratory tests were notable for negative human immunodeficiency virus (HIV) and two indeterminate QuantiFERON-TB test (Qiagen—Venlo, The Netherlands) results, along with marked leukocytosis. Chest CT showed an ill-defined mass in the right supra-hilar region with mediastinal invasion and pleural effusion (Figure 2). A lymph node biopsy was non-diagnostic, but bronchoscopy confirmed a diagnosis of *Mycobacterium avium* complex (MAC) on bronchial lavage. After starting treatment with clarithromycin 500 mg PO BID and ethambutol 15 mg/kg PO daily for 1 month, her leukocytosis was resolved but the patient did not have symptomatic improvement. Anti-IFN-γ AAb testing was pursued and returned positive (titer 10, 813) approximately two months following initial diagnosis with MAC. Treatment was changed to immunomodulatory therapy with rituximab 500 mg IV (375 mg/m^2^) administered weekly for four doses. The patient tolerated this well, reporting increasing energy, weight gain and significant symptom resolution. She was continued on clarithromycin and ethambutol as well for a total of 12 months. On recurrent follow-ups during this treatment period, symptoms continued to improve and there was also radiographic improvement on imaging without the need for ongoing anti-mycobacterial or biologic therapy.

**Figure 3:**
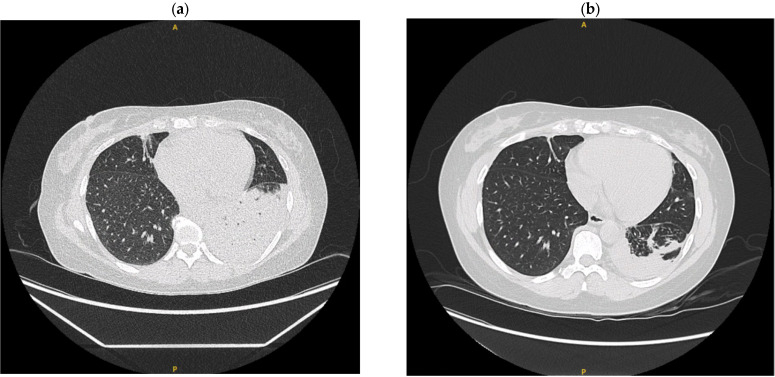
Evolution of chest CT imaging in Case 2: (**a**) before treatment—December 2023, (**b**) after treatment—April 2024. Improvement in left lower lobe consolidation with moderate left pleural effusion. A, anterior; P, posterior.

Case 2: A 45-year-old Canadian-born Filipino female with a history of systemic lupus erythematosus, childhood asthma and eczema on monoclonal antibody therapy with Dupilumab presented with acute-on-chronic dyspnea, cough, fever, and pleuritic chest pain. She had multiple similar presentations in the past 2 years, treated unsuccessfully with various antibiotics, bronchodilators and steroid trials. Laboratory findings included leukocytosis and elevated CRP. HIV testing was negative. CT imaging revealed persistent consolidation in the left lower lobe (Figure 3), with acid fast bacilli seen on biopsy from bronchoscopy with endobronchial ultrasound. Further testing revealed positivity for MAC on bronchial lavage and lymph node fine needle aspirate samples. After initiating triple therapy (rifampin 600 mg PO daily, ethambutol 15 mg/kg PO daily, and azithromycin 250 mg PO daily), she developed a rash attributed to rifampin, leading to a switch to dual therapy with ethambutol and macrolide (azithromycin, later switched to clarithromycin after hospital discharge). Anti-IFN-γ AAb testing was sent during admission and later returned positive (titer 16, 756). Approximately four months after the hospitalization, she received outpatient rituximab 600 mg IV (375 mg/m^2^) weekly for four doses. These were well tolerated, with only mild hives which were managed supportively. Leukocytosis normalized and there was notable improvement in the left lower lobe consolidation on CT chest. She was continued on anti-mycobacterial antibiotic therapy planned for a minimum of 18–24 months per last documented follow-up visit. The patient symptomatically reported feeling well at this time, and there have been no documented hospital readmissions in the health authority to date.

## 3. Discussion

Anti-IFN-γ AAbs are associated with an acquired adult-onset immunodeficiency syndrome often diagnosed after chronic and challenging infections emerge in previously healthy patients. This condition disrupts the immune response by interfering in IFN-γ signaling, which is critical for effective pathogen clearance. We describe below the current state of knowledge on the epidemiology of this condition and the mechanism of rituximab as a treatment option.

A study on anti-IFN-γ AAb immunodeficiency syndrome highlighted demographic and clinical differences between patient cohorts from Thailand and the United States (US) [10]. In the US, most affected individuals were Southeast Asian women, while the Thai group showed a more diverse demographic with fewer women. Female predominance ranged from 64% in the Thai population up to 91% in the US [10]. Infection patterns varied by region, with *Mycobacterium abscessus* being more common in Thailand and MAC being more prevalent in the US. The Thai cohort had a higher mortality rate (24%), largely due to severe, drug-resistant infections. Although initial AAb levels were similar in both groups, they naturally declined over time, aligning with reduced disease severity, even without targeted immunomodulatory treatment. While treatments like rituximab and cyclophosphamide helped reduce AAb levels, their impact on preventing infections remains unclear. The study suggests that monitoring AAb levels may aid in assessing infection risk and tracking disease progression. AAbs against IFN-γ are predominantly found in Southeast Asian populations, possibly due to genetic polymorphisms in the human leukocyte antigen (HLA) system; however, cases have also been documented in Japan, South Africa, Europe, South Asia, and the US [7,8,10,16]. Testing for anti-IFN-γ AAbs should be considered in cases of opportunistic infections without known immunocompromised conditions, including unexplained or recurrent or persistent NTM infections. For instance, one case involved an HIV patient with a well-controlled disease who developed dNTM and was later found to have anti-IFN-γ AAbs [17].

**Figure 4:**
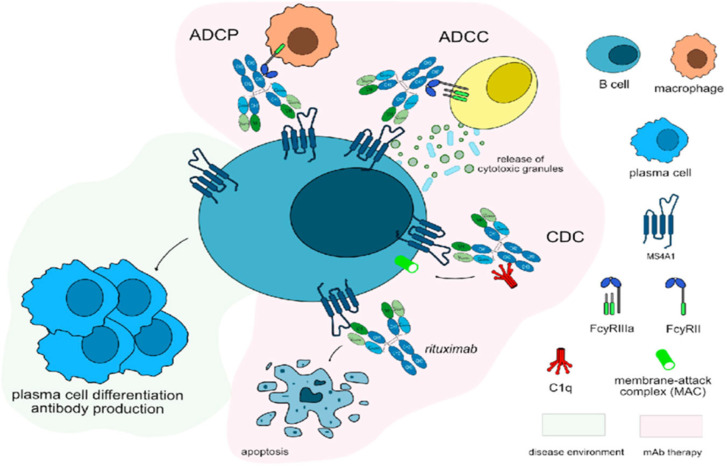
Rituximab mechanism of action. Rituximab targets and binds to MS4A1 expressed on the surface of B cells. Once bound to its target, rituximab induces apoptosis of CD20+ cells, resulting in depletion of B-cells. Rituximab is an IgG1-kappa antibody able to mediate complement-dependent cytotoxicity (CDC), antibody-dependent cellular cytotoxicity (ADCC) and antibody-dependent cellular phagocytosis (ADCP) against CD20+ B cells to completely deplete this population. Mechanism of action: Blocking. Effect: Immunosuppressant, Fc-effector function. (mAbID). Reprinted with permission from Golfinopoulou et al. [31]. Copyright 2023 by the authors.

Rituximab, a monoclonal antibody targeting the CD20 antigen on B cells, depletes circulating B cells, which may reduce anti-IFN-γ AAb levels and restore IFN-γ pathway activity (Figure 4). The potential benefit of rituximab lies in limiting further anti-IFN-γ AAb production, thus enabling an effective immune response alongside targeted antimicrobial therapy [18]. Doses are generally modeled after lymphoma protocols (375 mg/m^2^ intravenously, weekly for 4–8 weeks), with case reports documenting decreased anti-IFN-γ AAb titers and infection clearance following rituximab administration [19,20].

Rituximab is not currently included in the standard treatment recommendations for NTM-related pulmonary disease in the 2020 American Thoracic Society/Infectious Diseases Society of America (ATS/IDSA) guidelines [21]. Although typically contraindicated in patients with active infections, rituximab has shown efficacy as adjunctive therapy in cases with anti-IFN-γ AAbs, improving IFN-γ function and aiding infection resolution [22,23]. In certain complex cases involving coexisting immune-mediated conditions, such as granulomatosis with polyangiitis, rheumatoid arthritis, or interstitial lung diseases associated with autoimmune features, rituximab may be used off-label to control underlying inflammation that contributes to lung pathology or complicates infection management [24,25]. Furthermore, IFN-γ cytokine replacement therapies have limited effectiveness in the case of pathology in interferon gamma receptor subunit 1 (IFNGR1), a part of the JAK-STAT signaling pathway [26,27]. For patients with IFNGR1 deficiency or AAbs who are also at risk of chronic intracellular infections, rituximab may be a viable option.

In our case, rituximab was administered as part of a multidisciplinary decision based on the clinical context of progressive disease despite appropriate antimycobacterial therapy, and concurrent inflammatory lung disease that was contributing to radiologic and symptomatic worsening. Although B-cell depletion with rituximab is associated with increased risk of infections, including reactivation of latent infections and opportunistic pathogens such as *Pneumocystis jirovecii* (PJP) and Hepatitis B virus [28,29], the risk–benefit balance favored immunomodulation to control the underlying inflammatory process in our patient clinical trajectories. Close microbiological monitoring and prophylactic measures (e.g., PJP prophylaxis) were undertaken to mitigate these risks. A recent review emphasizes the importance of individualized risk assessment, interdisciplinary collaboration, and careful patient selection when considering rituximab in infectious contexts [30].

## 4. Conclusions

In summary, patients with anti-IFN-γ AAbs often suffer from recurrent infections like progressive dNTM, deep-seated mycoses and even chronic recurrent bacterial infections that are unresponsive to standard antimicrobial therapy. The IFN-γ and IL-12 pathways play essential roles in immunity against intracellular pathogens, underscoring the need for pathway evaluations in patients with severe or resistant NTM infections after ruling out other immunodeficiencies like HIV. Treatment is challenging due to delayed diagnosis and the absence of standardized protocols. Early recognition and testing for anti- IFN-γ AAbs may improve patient outcomes in addition to directed antimicrobial therapy. Rituximab may have a role in the therapy of treatment-refractory cases, though individualized infectious risk assessment should be completed prior to initiation.

## Data Availability

The data presented in this article is not readily available for protection of patient confidentiality. Requests for access can be directed to the corresponding author.
